# Association of novel inflammatory biomarkers with major amputation level in diabetic foot ulcer patients undergoing amputation: a retrospective comparative study

**DOI:** 10.3389/fendo.2026.1925839

**Published:** 2026-09-21

**Authors:** Hua Chen, Xiongliang Zhang, Jiezhi Dai

**Affiliations:** Department of Orthopedic Surgery, Shanghai Sixth People’s Hospital, JiaoTong University, Shanghai, China

**Keywords:** above-knee amputation, below-knee amputation, diabetic foot ulcer, inflammation-related biomarkers, LASSO regression, major amputation, RAR

## Abstract

**Background:**

This study aimed to comprehensively evaluate the association of twelve inflammation-related biomarkers with amputation level (major vs. minor) in diabetic foot ulcer (DFU) patients undergoing amputation, with particular focus on identifying the strongest associated factor and exploring differences between above-knee amputation (AKA) and below-knee amputation (BKA).

**Methods:**

We retrospectively enrolled DFU patients who underwent amputation between January 2020 and June 2025. Twelve indices were calculated: SIRI, SII, AISI, NAR, MAR, RAR, HALP, PNI, ALI, CALLY, IBI, and CLR. LASSO regression and multivariate logistic regression were used for variable selection. AUC comparisons and Bootstrap internal validation assessed model performance. Subgroup analysis compared AKA vs. BKA.

**Results:**

A total of 538 patients were included (271 major, 267 minor). In univariate analysis, higher levels of NAR, MAR, RAR, SIRI, SII, AISI, IBI, and CLR were associated with major amputation, whereas lower levels of HALP, PNI, ALI, and CALLY were associated with major amputation. RAR exhibited the highest odds ratio (OR = 3.25, 95% CI: 2.54-4.16, p < 0.001) for major versus minor amputation. LASSO regression selected eight indices; multivariate analysis confirmed RAR, HALP, PNI, and ALI as independently associated with amputation level. The combined model incorporating RAR, HALP, PNI, and ALI demonstrated superior predictive performance compared to RAR alone (AUC: 0.816 vs. 0.773, p = 0.001). Bootstrap validation confirmed the stability of the combined model (optimism-corrected AUC: 0.811; optimism: 0.005). In the exploratory subgroup analysis, AKA patients had significantly higher RAR levels and lower PNI and ALI levels compared to BKA patients (all p < 0.05); however, only RAR remained significantly associated with AKA after multivariable adjustment.

**Conclusion:**

The combined model may provide modest incremental predictive value over RAR alone, but the clinical gain is limited. RAR alone remains a practical and sufficient tool for risk stratification, particularly in resource-limited settings. However, these findings are retrospective, and external validation is warranted before clinical application.

## 1 Background

Diabetic foot ulcer (DFU) is one of the most devastating complications of diabetes mellitus, affecting approximately 15-25% of diabetic patients during their lifetime (1). Despite advances in multidisciplinary care, lower-extremity amputation remains a common outcome, with major amputation (above the ankle) carrying a dismal prognosis-five-year mortality rates exceeding 50%-and profoundly affecting quality of life (2). Early risk assessment of the risk of amputation in DFU patients can reduce hospitalization, disability, and mortality rates.

Hematological markers are widely used to assess immune function, nutritional status, and inflammation (3). As a routine and cost-effective laboratory test, complete blood count (CBC) provides valuable data on blood components. The absolute counts of neutrophils, lymphocytes, monocytes, and platelets, along with derived parametric indices, can serve as valuable systemic inflammatory biomarkers. In addition, other biomarkers, such as CRP and albumin, can also reflect systemic inflammation (4, 5). Recently, novel composite indicators combining CBC, CRP, and albumin have been developed, demonstrating superior performance over single parameters in predicting outcomes in various diseases (6).

Accumulating evidence has demonstrated the utility of inflammation-related biomarkers in evaluating diabetes progression and its associated complications. In two cross-sectional analyses of the 1999–2004 NHANES database, NAR, MAR, RAR, HALP, PNI, SIRI, SII, and AISI were significantly associated with the prevalence of DFU (7, 8). Through a cross-sectional analysis of the 1999–2018 NHANES database, higher ALI values were found to be significantly correlated with a decreased prevalence of diabetic retinopathy (9). Peng et al. reported that CALLY index was associated with hypertension prevalence in diabetic adults (10). Yu et al. observed a stronger relationship between pre-diabetes, diabetes mellitus, and IBI in the U.S. population (11). Despite these advances, the application of these biomarkers in distinguishing major from minor amputation in patients with DFU who have undergone amputation has not been adequately investigated. This study, based on data from a single hospital, is the first to evaluate the association between several inflammation-related biomarkers and the amputation level in patients with DFU undergoing amputation.

## 2 Methods

This retrospective study was approved by the Ethics Committee of shanghai sixth peoples’ hospital (approval no. 2025-ky-198(K)) and was conducted in accordance with the Declaration of Helsinki. The requirement for written informed consent was waived by the Ethics Committee due to the retrospective nature of the study and the use of anonymized data.

We reviewed medical records of consecutive DFU patients who underwent lower-extremity amputation between January 2020 and Jun 2025. Inclusion criteria: (1) age ≥ 18 years; (2) diagnosis of type 1 or type 2 diabetes mellitus; (3) diabetic foot ulcer classified according to the International Working Group on the Diabetic Foot (IWGDF); (4) underwent unilateral amputation. Exclusion criteria: (1) active malignancy; (2) severe systemic infection requiring intensive care unit (ICU) admission at presentation, defined as infection associated with hemodynamic instability, sepsis, or septic shock as per the Sepsis-3 criteria (12). Localized foot infections without systemic involvement were not excluded; (3) missing values for the inflammation-related biomarkers; (4) incomplete laboratory data.

### 2.1 Data collection

Demographic characteristics and laboratory parameters were extracted from electronic medical records.

SIRI = Neutrophil (×10^9^/L) × Monocyte (×10^9^/L)/Lymphocyte (×10^9^/L)

SII = Platelet (×10^9^/L) × Neutrophil (×10^9^/L)/Lymphocyte (×10^9^/L)

AISI = Neutrophil × Monocyte × Platelet/Lymphocyte

NAR = Neutrophil/Albumin (g/L)

MAR = Monocyte/Albumin

RAR = Red cell distribution width (%)/Albumin

HALP score = Hemoglobin (g/L) × Albumin (g/L) × Lymphocyte (×10^9^/L)/Platelet (×10^9^/L)

PNI = Albumin (g/L) + 5 × Lymphocyte (×10^9^/L)

ALI = BMI × Albumin/(Neutrophil/Lymphocyte)

CALLY = (Albumin × Lymphocyte)/CRP (mg/L)

IBI = CRP (mg/L) × Neutrophil/Lymphocyte

CLR = CRP (mg/L)/Lymphocyte

To compare the relative strength of association among different inflammatory indices, continuous variables were standardized to Z-scores (mean = 0, SD = 1) before entry into the multivariate logistic regression model. The resulting odds ratios represent the change in risk of major amputation per one standard deviation increase in the respective index.

### 2.2 Study design

In this study, patients were divided into two groups based on their amputation status: the first group included those who underwent minor amputation: amputation below the ankle (toe, ray, transmetatarsal, or midfoot amputation); and the second group included those who underwent major amputation: amputation at or above the ankle joint (below-knee, through-knee, or above-knee amputation). In patients who underwent multiple amputations during hospitalization due to poor wound healing and infection, the final (most proximal) level of amputation was used for analysis.

At our institution, amputation level decisions were made by a multidisciplinary team following a standardized protocol based on the WIfI classification. WIfI stage ≥ 4 generally indicated major amputation, while stage ≤ 3 was considered for limb salvage when feasible. Perfusion was assessed by ABI; ABI < 0.5 indicated severe ischemia, warranting revascularization evaluation to preserve below-knee potential. Above knee amputation was reserved for cases where revascularization was not feasible or infection/necrosis precluded a functional below-knee level. Infection severity was graded by PEDIS (grade ≥ 3 favoring major amputation), and functional status (community/household/non-ambulator) also informed the decision. Final decisions were reached by multidisciplinary consensus.

### 2.3 Statistical analysis

All analyses were performed using SPSS version 18.0, R version 4.3.3, and Zstats 1.0 (https://www.zstats.net). A two-sided p < 0.05 was considered statistically significant.

Continuous variables were presented as mean ± standard deviation or median (interquartile range) based on normality, and categorical variables as frequencies (%). Comparisons were made using Student’s t-test, Mann-Whitney U test, or chi-square test, as appropriate. Missing data were handled as follows: for categorical variables, missing values were imputed using the most frequent category; for continuous variables, missing values were imputed using mean imputation for normally distributed variables and median imputation for non-normally distributed variables. The proportion of missing values for each variable was < 5%.

Serum levels of inflammatory indices were divided into tertiles, with the lowest tertile (Q1) serving as the reference group. Univariate logistic regression was first performed to evaluate the association between each inflammatory biomarker and major versus minor amputation. Subsequently, three multivariate binary logistic regression models were established: Model 1 (unadjusted), Model 2 (adjusted for age and sex), and Model 3 (adjusted for age, sex, FBG, BMI, hypertension, and diabetes duration). To explore potential nonlinear relationships, restricted cubic spline (RCS) curves and threshold effect analyses were conducted.

To address multicollinearity among the twelve inflammation-related biomarkers, we first performed LASSO regression with 10-fold cross-validation to select the most parsimonious set of predictors. Biomarkers with non-zero coefficients at the optimal λ (λ.min) were retained as candidate variables. These candidates were then entered into a multivariate binary logistic regression model adjusting for clinically relevant confounders (age, sex, FBG, BMI, hypertension, and diabetes duration). This two-step approach was used to identify factors associated with amputation level while minimizing the risk of false-positive findings due to collinearity or confounding.

The discriminative ability of each inflammatory biomarker for major versus minor amputation was assessed using receiver operating characteristic (ROC) curves, with the area under the curve (AUC) reported. Optimal cut-off values were determined by maximizing the Youden index (sensitivity + specificity - 1). To evaluate the stability of the cut-off value, bootstrap resampling with 1,000 iterations was performed, and bootstrap-derived median cut-offs with 95% percentile confidence intervals were calculated.

To compare model performance, RAR alone and the LASSO-multivariate-selected model (the combined model) were evaluated using AUC analysis with DeLong tests. Decision curve analysis (DCA) was performed to assess the clinical net benefit of the models across a range of threshold probabilities. Given the exploratory nature of this study, internal validation was performed using bootstrap resampling (200 resamples) to evaluate the potential for overfitting. The optimism-corrected C-statistic was derived from the validation process. Model calibration was assessed via calibration curves with intercept and slope correction, and overall model performance was evaluated using the Brier score.

For the subgroup analysis comparing AKA and BKA, we first compared all twelve indices between the two groups using Mann-Whitney U tests or Student’s t-test. To minimize overfitting given the limited sample size (AKA, n = 55), a parsimonious multivariable logistic regression model was then performed to identify indices associated with AKA (with BKA as the reference). Based on the univariate findings and clinical relevance, the model included RAR, PNI, ALI, age, and sex (5 variables), which satisfied the rule of at least 10 events per variable (55 events/5 variables = 11 events per variable). Finally, ROC analysis was conducted to evaluate the discriminative ability of significant indices in distinguishing AKA from BKA. Given the limited sample size in the AKA group, this analysis was pre-specified as exploratory. Accordingly, results should be interpreted as hypothesis-generating, and no adjustment for multiple comparisons was applied to avoid inflating type II error, consistent with the exploratory nature of this analysis.

## 3 Results

A total of 538 patients who underwent amputation to DFU are included in this study (**Table 1**). Overview of research process and study design were presented in **Figure 1**. The mean age of the patients was 65.31 ± 10.73 years old and 117 (21.75%) were female. Minor amputation were performed in 267 patients and major amputation in 271 patients. Among the 271 patients in the major amputation group, 20 patients (7.4%) underwent staged amputation. Patients in major amputation group exhibited elevated levels of CRP, WBCs, monocytes, neutrophils, platelets, RDW, and FBG, with lower levels of hemoglobin, lymphocytes, albumin, and HbA1c. Regarding the inflammation-related biomarkers, patients with major amputation had higher levels of SIRI, SII, AISI, NAR, MAR, RAR, IBI, CLR, and lower levels of HALP, PNI, ALI, and CALLY.

**Table 1:** Clinical characteristics of the retrospective study.

Characteristics	Total	Minor amputation (n=267)	Major amputation (n=271)	p
Age (years)	65.31 ± 10.73	65.03 ± 10.69	65.59 ± 10.79	0.551
Gender				
Male	421	219	202	0.035
Female	117	48	69	
Hypertension				
Yes	330	181	149	0.002
No	208	86	122	
BMI (kg/m2)	22.83 ± 3.20	23.29 ± 2.92	22.37 ± 3.40	<0.001
Duration of diabetes (years)	18.72 ± 4.30	18.51 ± 4.14	18.93 ± 4.45	0.261
CRP (mg/L)	66.50 ± 59.12	51.54 ± 54.36	81.24 ± 60.02	<0.001
Hb (g/L)	101.01 ± 22.64	111.05 ± 20.47	91.11 ± 20.21	<0.001
WBC (10^9^)	11.63 ± 6.28	9.67 ± 4.60	13.55 ± 7.07	<0.001
Lymphocyte (10^9^)	1.42 ± 0.59	1.49 ± 0.54	1.34 ± 0.62	0.004
Monocyte (10^9^)	0.76 ± 0.37	0.71 ± 0.30	0.82 ± 0.42	<0.001
Neutrophil (10^9^)	9.16 ± 5.92	7.23 ± 4.37	11.06 ± 6.60	<0.001
Platelet (10^9^)	315.14 ± 121.38	286.86 ± 103.05	343.00 ± 131.42	<0.001
RDW (%)	13.87 ± 2.11	13.15 ± 1.74	14.59 ± 2.21	<0.001
Albumin (g/L)	31.55 ± 5.44	33.44 ± 5.11	29.68 ± 5.12	<0.001
FBG (mmol/L)	9.40 ± 4.15	8.99 ± 4.04	9.80 ± 4.22	0.023
HbAlc (%)	8.70 ± 2.03	8.92 ± 2.01	8.48 ± 2.04	0.012
NAR	2.35 (1.53, 3.92)	1.71 (1.24, 2.86)	3.15 (2.02, 5.04)	<0.001
MAR	0.22 (0.16, 0.30)	0.19 (0.14, 0.27)	0.25 (0.18, 0.34)	<0.001
RAR	4.25 (3.70, 5.24)	3.88 (3.45, 4.33)	4.89 (4.15, 5.81)	<0.001
HALP	13.82 (8.33, 23.67)	19.01 (11.84, 30.58)	9.85 (6.35, 15.70)	<0.001
PNI	39.00 (34.12, 43.30)	41.30 (36.50, 45.00)	36.50 (31.50, 41.00)	<0.001
ALI	12.90 (6.69, 22.03)	17.66 (10.76, 28.11)	8.43 (4.56, 14.76)	<0.001
SIRI	3.99 (2.01, 8.18)	2.85 (1.56, 5.47)	5.32 (2.93, 11.56)	<0.001
SII	1714.93 (879.23, 3316.50)	1178.60 (711.32, 1942.72)	2592.87 (1381.57, 4365.02)	<0.001
AISI	1158.41 (544.02, 2632.82)	721.29 (387.34, 1625.98)	1826.25 (873.27, 3631.12)	<0.001
CALLY	0.86 (0.34, 2.80)	1.52 (0.51, 5.69)	0.54 (0.27, 1.21)	<0.001
IBI	279.14 (66.97, 922.18)	123.28 (27.83, 505.16)	509.58 (178.11, 1374.75)	<0.001
CLR	37.95 (11.09, 85.87)	21.48 (5.97, 63.57)	56.95 (24.70, 98.15)	<0.001

RDW, red cell distribution width; FBG, Fasting Blood Glucose; NAR, neutrophil-albumin ratio; MAR, monocyte-albumin ratio; RAR, red cell distribution width-albumin ratio; HALP, hemoglobin, albumin, lymphocyte, and platelet; PNI, prognostic nutritional index; ALI, advanced lung cancer inflammation index; SIRI, systemic inflammatory response index; SII, systemic immune-inflammatory index; AISI, aggregate index of systemic inflammation; CALLY, C-reactive protein-albumin-lymphocyte index; IBI, inflammatory burden index; CLR, C-reactive protein to lymphocyte ratio.

**Figure 1:**
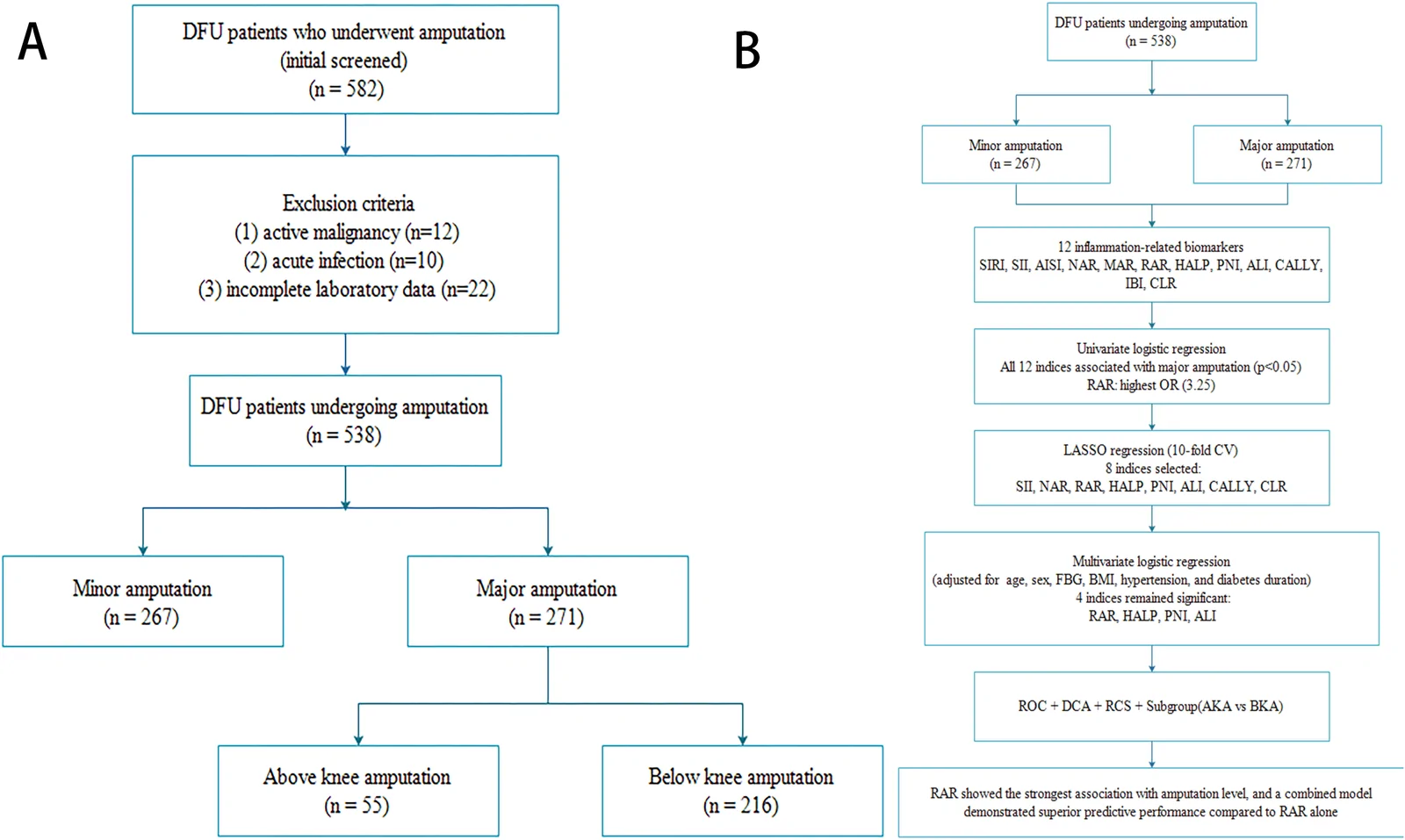
Overview of research process and design. **(A)** Patient selection flowchart; **(B)** Study design diagram.

### 3.1 Association of twelve inflammation-related biomarkers with major versus minor amputation in logistic regression analysis

Univariate logistic regression identified all twelve indices as significantly associated with major versus minor amputation (all p < 0.05). Among these, RAR exhibited the highest odds ratio (OR = 3.25, 95% CI: 2.54-4.16, p < 0.001), indicating the strongest individual association (**Figure 2**). When analyzed in continuous form, a significant correlation between SIRI, SII, AISI, NAR, MAR, RAR, HALP, PNI, ALI, CALLY, IBI, CLR and amputation level was observed in the unadjusted model 1, adjusted model 2, and adjusted model 3 (**Table 2**). After multivariable adjustment (Model 3), RAR remained the strongest independent correlate (OR = 3.18, 95% CI: 2.47-4.10, p < 0.001) (**Table 2**). E-value analysis for RAR (adjusted OR = 3.18) yielded an E-value of 5.81, indicating robustness against unmeasured confounding.

**Figure 2:**
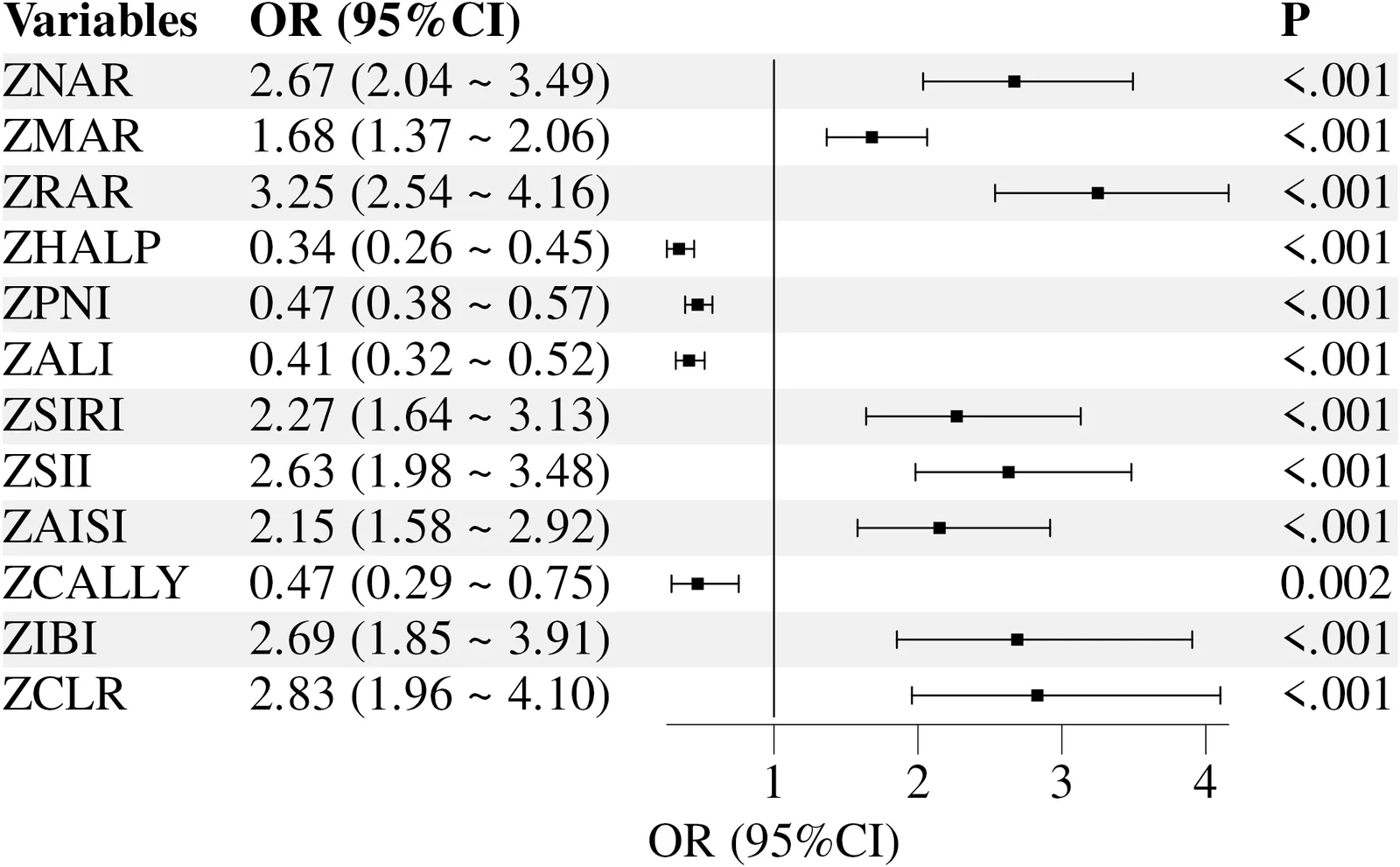
Forest plot of multivariable regression.

**Table 2:** Association of inflammation-related biomarkers with major versus minor amputation in logistic regression analysis.

Variable	Model 1	P	Model 2	P	Model 3	P
	OR (95%CI)		OR (95%CI)		OR (95%CI)	
ZNAR	2.67 (2.04 ~ 3.49)	<0.001	2.72 (2.07 ~ 3.58)	<0.001	2.69 (2.03 ~ 3.56)	<0.001
ZNAR tertiles						
Tertiles 1	1.00 (Reference)	–	1.00 (Reference)	–	1.00 (Reference)	–
Tertiles 2	3.53 (2.26 ~ 5.51)	<0.001	3.50 (2.23 ~ 5.49)	<0.001	3.75 (2.36 ~ 5.98)	<0.001
Tertiles 3	7.36 (4.61 ~ 11.76)	<0.001	7.61 (4.73 ~ 12.22)	<0.001	7.74 (4.72 ~ 12.71)	<0.001
*P*-trend		<0.001		<0.001		<0.001
ZMAR	1.68 (1.37 ~ 2.06)	<0.001	1.75 (1.41 ~ 2.16)	<0.001	1.78 (1.43 ~ 2.22)	<0.001
ZMAR tertiles						
Tertiles 1	1.00 (Reference)	–	1.00 (Reference)	–	1.00 (Reference)	–
Tertiles 2	1.67 (1.10 ~ 2.55)	0.017	1.74 (1.13 ~ 2.67)	0.012	1.82 (1.17 ~ 2.84)	0.008
Tertiles 3	3.12 (2.02 ~ 4.81)	<0.001	3.49 (2.23 ~ 5.45)	<0.001	3.61 (2.27 ~ 5.73)	<0.001
*P*-trend		<0.001		<0.001		<0.001
ZRAR	3.25 (2.54 ~ 4.16)	<0.001	3.25 (2.53 ~ 4.17)	<0.001	3.18 (2.47 ~ 4.10)	<0.001
ZRAR tertiles						
Tertiles 1	1.00 (Reference)		1.00 (Reference)		1.00 (Reference)	
Tertiles 2	3.45 (2.18 ~ 5.45)	<0.001	3.38 (2.14 ~ 5.35)	<0.001	3.39 (2.13 ~ 5.42)	<0.001
Tertiles 3	12.47 (7.57 ~ 20.56)	<0.001	12.49 (7.54 ~ 20.68)	<0.001	11.83 (7.03 ~ 19.91)	<0.001
*P*-trend		<0.001		<0.001		<0.001
ZHALP	0.34 (0.26 ~ 0.45)	<0.001	0.34 (0.26 ~ 0.45)	<0.001	0.36 (0.28 ~ 0.48)	<0.001
ZHALP tertiles						
Tertiles 1	1.00 (Reference)		1.00 (Reference)		1.00 (Reference)	
Tertiles 2	0.28 (0.18 ~ 0.44)	<0.001	0.29 (0.18 ~ 0.45)	<0.001	0.29 (0.18 ~ 0.46)	<0.001
Tertiles 3	0.10 (0.06 ~ 0.17)	<0.001	0.11 (0.07 ~ 0.17)	<0.001	0.12 (0.07 ~ 0.19)	<0.001
*P*-trend		<0.001		<0.001		<0.001
ZPNI	0.47 (0.38 ~ 0.57)	<0.001	0.47 (0.39 ~ 0.58)	<0.001	0.50 (0.41 ~ 0.61)	<0.001
ZPNI tertiles						
Tertiles 1	1.00 (Reference)		1.00 (Reference)		1.00 (Reference)	
Tertiles 2	0.60 (0.39 ~ 0.92)	0.020	0.61 (0.39 ~ 0.93)	0.022	0.66 (0.42 ~ 1.02)	0.060
Tertiles 3	0.24 (0.15 ~ 0.37)	<0.001	0.24 (0.15 ~ 0.38)	<0.001	0.28 (0.18 ~ 0.45)	<0.001
*P*-trend		<0.001		<0.001		<0.001
ZALI	0.41 (0.32 ~ 0.52)	<0.001	0.41 (0.32 ~ 0.52)	<0.001	0.42 (0.32 ~ 0.54)	<0.001
ZALI tertiles						
Tertiles 1	1.00 (Reference)		1.00 (Reference)		1.00 (Reference)	
Tertiles 2	0.34 (0.22 ~ 0.53)	<0.001	0.33 (0.21 ~ 0.52)	<0.001	0.36 (0.23 ~ 0.56)	<0.001
Tertiles 3	0.12 (0.08 ~ 0.20)	<0.001	0.12 (0.08 ~ 0.20)	<0.001	0.13 (0.08 ~ 0.22)	<0.001
*P*-trend		<0.001		<0.001		<0.001
ZSIRI	2.27 (1.64 ~ 3.13)	<0.001	2.31 (1.66 ~ 3.21)	<0.001	2.29 (1.63 ~ 3.22)	<0.001
ZSIRI tertiles						
Tertiles 1	1.00 (Reference)		1.00 (Reference)		1.00 (Reference)	
Tertiles 2	2.02 (1.32 ~ 3.09)	0.001	2.03 (1.32 ~ 3.12)	0.001	2.13 (1.36 ~ 3.35)	<0.001
Tertiles 3	4.28 (2.75 ~ 6.67)	<0.001	4.37 (2.80 ~ 6.83)	<0.001	4.65 (2.90 ~ 7.47)	<0.001
*P*-trend		<0.001		<0.001		<0.001
ZSII	2.63 (1.98 ~ 3.48)	<0.001	2.63 (1.98 ~ 3.49)	<0.001	2.52 (1.89 ~ 3.36)	<0.001
ZSII tertiles						
Tertiles 1	1.00 (Reference)		1.00 (Reference)		1.00 (Reference)	
Tertiles 2	2.23 (1.44 ~ 3.45)	<0.001	2.17 (1.40 ~ 3.36)	<0.001	2.20 (1.40 ~ 3.45)	<0.001
Tertiles 3	7.58 (4.74 ~ 12.14)	<0.001	7.54 (4.70 ~ 12.09)	<0.001	7.53 (4.59 ~ 12.36)	<0.001
*P*-trend		<0.001		<0.001		<0.001
ZAISI	2.15 (1.58 ~ 2.92)	<0.001	2.18 (1.60 ~ 2.98)	<0.001	2.09 (1.53 ~ 2.86)	<0.001
ZAISI tertiles						
Tertiles 1	1.00 (Reference)		1.00 (Reference)		1.00 (Reference)	
Tertiles 2	2.20 (1.43 ~ 3.38)	<0.001	2.17 (1.41 ~ 3.35)	<0.001	2.28 (1.45 ~ 3.57)	<0.001
Tertiles 3	5.88 (3.72 ~ 9.29)	<0.001	6.02 (3.79 ~ 9.54)	<0.001	6.26 (3.86 ~ 10.16)	<0.001
*P*-trend		<0.001		<0.001		<0.001
ZCALLY	0.47 (0.29 ~ 0.75)	0.002	0.48 (0.30 ~ 0.77)	0.002	0.50 (0.31 ~ 0.80)	0.004
ZCALLY tertiles						
Tertiles 1	1.00 (Reference)		1.00 (Reference)		1.00 (Reference)	
Tertiles 2	0.52 (0.34 ~ 0.81)	0.003	0.51 (0.33 ~ 0.78)	0.002	0.53 (0.34 ~ 0.83)	0.005
Tertiles 3	0.19 (0.12 ~ 0.30)	<0.001	0.19 (0.12 ~ 0.30)	<0.001	0.20 (0.13 ~ 0.32)	<0.001
*P*-trend		<0.001		<0.001		<0.001
ZIBI	2.69 (1.85 ~ 3.91)	<0.001	2.71 (1.86 ~ 3.95)	<0.001	2.65 (1.80 ~ 3.90)	<0.001
ZIBI tertiles						
Tertiles 1	1.00 (Reference)		1.00 (Reference)		1.00 (Reference)	
Tertiles 2	3.21 (2.07 ~ 4.98)	<0.001	3.12 (2.01 ~ 4.86)	<0.001	3.13 (1.99 ~ 4.95)	<0.001
Tertiles 3	5.74 (3.63 ~ 9.05)	<0.001	5.81 (3.67 ~ 9.19)	<0.001	5.99 (3.69 ~ 9.74)	<0.001
*P*-trend		<0.001		<0.001		<0.001
ZCLR	2.83 (1.96 ~ 4.10)	<0.001	2.86 (1.98 ~ 4.15)	<0.001	2.82 (1.92 ~ 4.13)	<0.001
ZCLR tertiles						
Tertiles 1	1.00 (Reference)		1.00 (Reference)		1.00 (Reference)	
Tertiles 2	3.23 (2.09 ~ 5.00)	<0.001	3.11 (2.01 ~ 4.83)	<0.001	3.07 (1.96 ~ 4.82)	<0.001
Tertiles 3	4.42 (2.83 ~ 6.91)	<0.001	4.42 (2.83 ~ 6.92)	<0.001	4.33 (2.71 ~ 6.93)	<0.001
*P*-trend		<0.001		<0.001		<0.001

Model 1: Crude; Model 2: Adjust: Sex, Age; Model3: Adjust: Sex, Age, FBG, BMI, diabetes duration, and hypertension. NAR, neutrophil-albumin ratio; MAR, monocyte-albumin ratio; RAR, red cell distribution width-albumin ratio; HALP, hemoglobin, albumin, lymphocyte, and platelet; PNI, prognostic nutritional index; ALI, advanced lung cancer inflammation index; SIRI, systemic inflammatory response index; SII, systemic immune-inflammatory index; AISI, aggregate index of systemic inflammation; CALLY, C-reactive protein-albumin-lymphocyte index; IBI, inflammatory burden index; CLR, C-reactive protein-to-lymphocyte ratio.

We divided SIRI, SII, AISI, NAR, MAR, RAR, HALP, PNI, ALI, CALLY, IBI, and CLR into tertiles. In multivariate analysis adjusting for age, sex, FBG, BMI, hypertension, and diabetes duration, the odds for major amputation among those in the third tertile compared with those in the first tertile was increased by 1083% (OR = 11.83 [7.03-19.91], p < 0.001) for RAR, followed by 674% (OR = 7.74 [4.72-12.71], p < 0.001) for NAR, and 653% (OR = 7.53 [4.59-12.36], p < 0.001) for SII. The odds of major amputation among those in the third tertile compared with those in the first tertile was decreased by 88% (OR = 0.12 [0.07-0.19], p < 0.001) for HALP and 87% (OR = 0.13 [0.08-0.22], p < 0.001) for ALI (**Table 2**).

### 3.2 LASSO regression and multivariate analysis

Given the potential multicollinearity among variables and to prevent overfitting, LASSO regression with tenfold cross-validation was employed for variable selection. We selected λ = λ_min (0.0079) as it yielded the lowest prediction error, although it resulted in a slightly less sparse model compared to λ_1se. The LASSO coefficient paths (**Figure 3**) showed that SII, NAR, RAR, HALP, PNI, ALI, CALLY, and CLR had the largest coefficient magnitudes and were among the last variables to be shrunk to zero as λ increased, indicating their strong association with major versus minor amputation.

**Figure 3:**
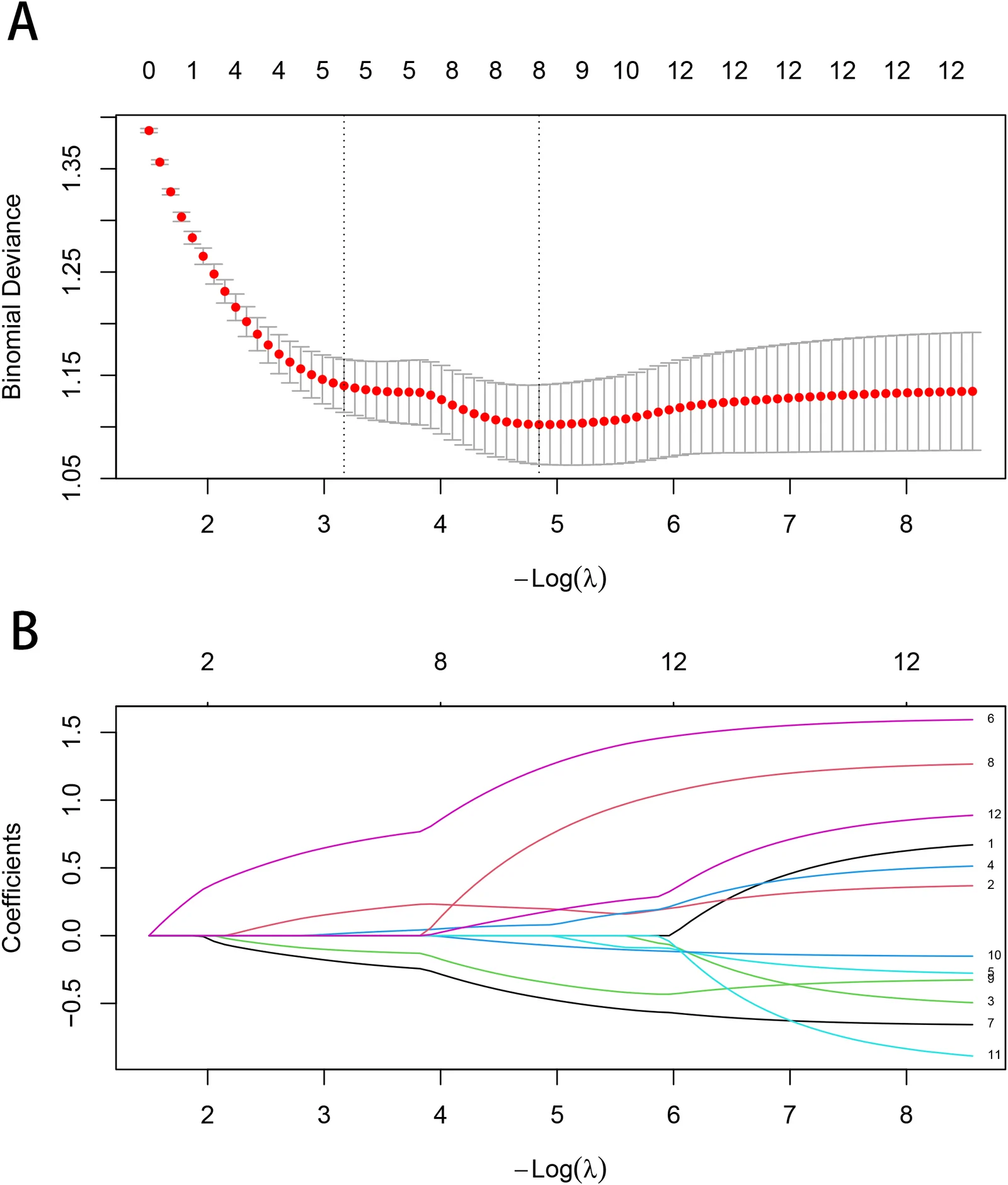
**(A)** All parameters were included in the LASSO analysis and coefficient curves were plotted. **(B)** Binomial deviations were plotted using the LASSO binary logistic regression model, which ultimately showed that 8 parameters were significant.

The eight variables identified by LASSO regression were included in the multivariate logistic regression model. Multivariate logistic regression adjusting for age, sex, FBG, BMI, hypertension, and diabetes duration, demonstrated that RAR (OR = 5.247, 95% CI: 3.335-8.254, p < 0.001) and PNI (OR = 3.718, 95% CI: 2.279-6.065, p < 0.001) were associated with major amputation level, whereas HALP (OR = 0.531, 95% CI: 0.366-0.771, p = 0.001), ALI (OR = 0.637, 95% CI: 0.424-0.957, p = 0.030) were associated with minor amputation (**Table 3**).

**Table 3:** Factors independently associated with major versus minor amputation identified by multivariate logistic regression analysis.

Variable	β coefficient	Standard error	P value	OR	95% CI OR	
					Lower	Upper
Hypertension	0.599	0.226	0.008	1.820	1.168	2.836
Sex	-0.167	0.266	0.530	0.846	0.502	1.426
AGE	0.017	0.010	0.097	1.017	0.997	1.038
FBG	0.039	0.027	0.157	1.040	0.985	1.097
BMI	-0.012	0.035	0.736	0.988	0.922	1.059
Diabetes duration	0.022	0.025	0.388	1.022	0.973	1.073
ZSII	0.121	0.231	0.601	1.128	0.718	1.774
ZRAR	1.658	0.231	<0.001	5.247	3.335	8.254
ZNAR	0.135	0.240	0.574	1.145	0.715	1.834
ZHALP	-0.632	0.190	0.001	0.531	0.366	0.771
ZPNI	1.313	0.250	<0.001	3.718	2.279	6.065
ZALI	-0.451	0.208	0.030	0.637	0.424	0.957
ZCALLY	-0.138	0.176	0.433	0.871	0.617	1.230
ZCLR	0.415	0.250	0.097	1.514	0.927	2.471
Constant	-1.622	1.245	0.193	0.198		

SII, systemic immune-inflammatory index; NAR, neutrophil-albumin ratio; RAR, red cell distribution width-albumin ratio; HALP, hemoglobin, albumin, lymphocyte, and platelet; PNI, prognostic nutritional index; ALI, advanced lung cancer inflammation index; CALLY, C-reactive protein-albumin-lymphocyte index; CLR, C-reactive protein to lymphocyte ratio.

### 3.3 Model comparison and predictive performance

We conducted an exploratory and comprehensive comparison of the discriminative ability of these twelve indices for major versus minor amputation (**Table 4**; **Figure 4**). Specifically, RAR showed the highest discriminative ability, with an AUC of 0.773. To assess the stability of the optimal cut-off values, we performed bootstrap resampling with 1,000 iterations for the primary biomarker (RAR). The bootstrap-adjusted median cut-off for RAR was 4.301 (95% CI: 4.005-4.408), confirming that the original cut-off (4.312) was relatively stable. We further plotted ROC curves and DeLong test to investigate whether combining these four indicators which identified by LASSO regression and multivariate logistic regression would provide better discriminative performance for major versus minor amputation compared to using a single indicator. The results showed that combined model yielded significantly higher AUC values compared to using a single indicator (AUC = 0.816 vs. 0.773, p = 0.001).

**Table 4:** Discriminative ability of 12 inflammation-related biomarkers for major versus minor amputation based on ROC analysis.

Variable	AUC	P	95%CI	Threshold	Sensitivity	Specificity
SIRI	0.682	<0.001	0.637-0.727	3.413	0.60	0.71
SII	0.733	<0.001	0.691-0.776	1728.576	0.697	0.686
AISI	0.704	<0.001	0.661-0.748	911.976	0.607	0.742
NAR	0.729	<0.001	0.687-0.771	2.197	0.652	0.723
MAR	0.644	<0.001	0.597-0.690	0.206	0.588	0.679
RAR	0.773	<0.001	0.734-0.813	4.312	0.745	0.705
HALP	0.746	<0.001	0.705-0.787	12.391	0.742	0.627
PNI	0.695	<0.001	0.651-0.739	39.050	0.633	0.657
ALI	0.734	<0.001	0.692-0.776	12.696	0.697	0.675
CALLY	0.695	<0.001	0.651-0.739	1.326	0.547	0.771
IBI	0.709	<0.001	0.666-0.753	194.440	0.596	0.738
CLR	0.681	<0.001	0.636-0.726	21.705	0.502	0.790

NAR, neutrophil-albumin ratio; MAR, monocyte-albumin ratio; RAR, red cell distribution width-albumin ratio; HALP, hemoglobin, albumin, lymphocyte, and platelet; PNI, prognostic nutritional index; ALI, advanced lung cancer inflammation index; SIRI, systemic inflammatory response index; SII, systemic immune-inflammatory index; AISI, aggregate index of systemic inflammation; CALLY, C-reactive protein-albumin-lymphocyte index; IBI, inflammatory burden index; CLR, C-reactive protein to lymphocyte ratio.

**Figure 4:**
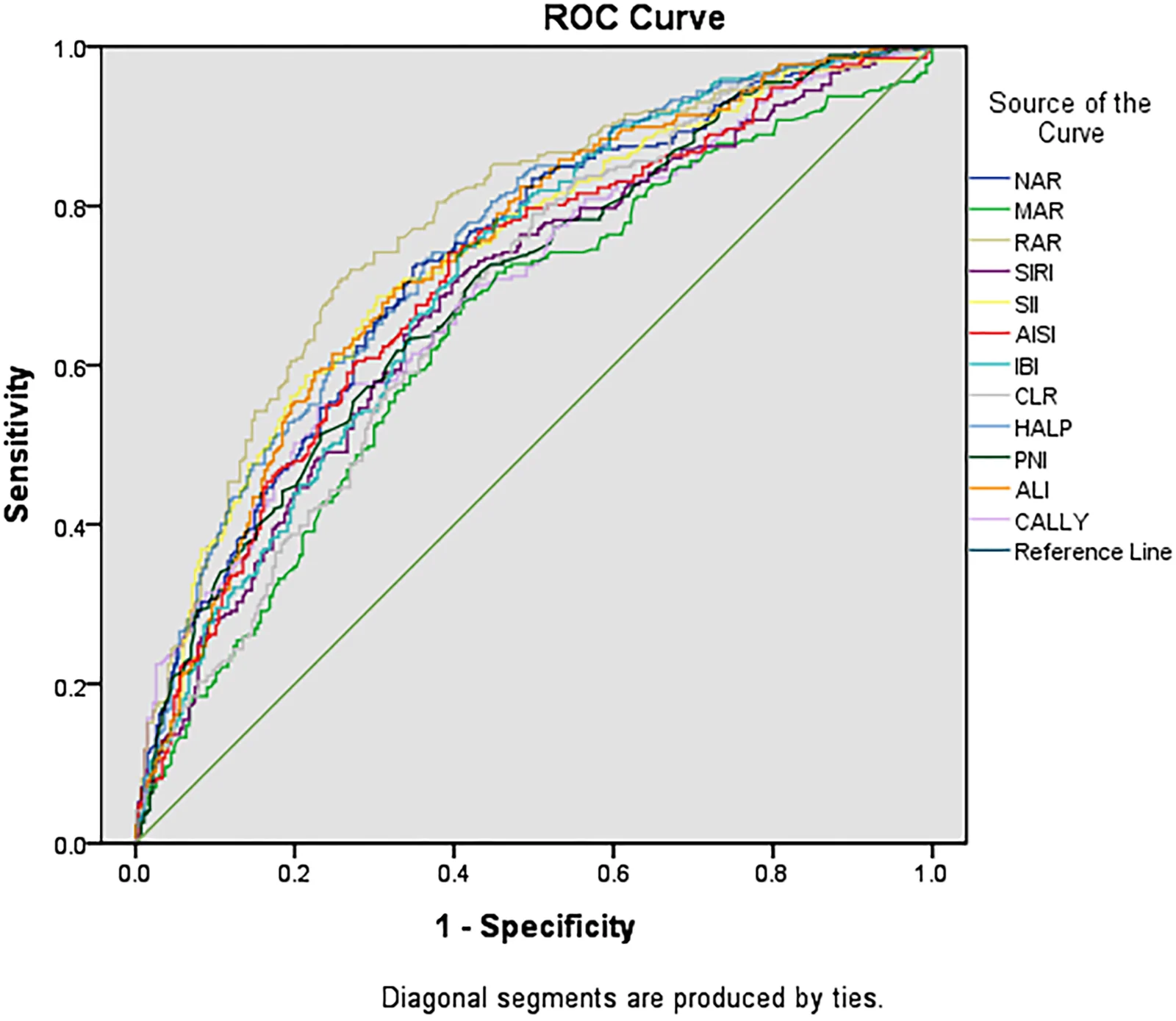
ROC curve evaluates the predictive performance of all twelve inflammation-related biomarkers for the risk of major amputation.

Bootstrap internal validation (200 resamples) demonstrated that both models were stable and well calibrated, with minimal optimism (combined model: 0.005; RAR alone: 0.000). The optimism-corrected C-statistic for the combined model was 0.811 (apparent: 0.816), with a calibration intercept of 0.002 and slope of 0.964. For the RAR-alone model, the optimism-corrected C-statistic was 0.774 (apparent: 0.773), with a calibration intercept of -0.001 and slope of 1.004. The Brier scores were 0.176 and 0.196, respectively. The calibration plot showed that the bias-corrected curve remained close to the ideal line, confirming that the model was well calibrated and not substantially overfitted (**Supplementary Figure S1**). These findings confirm that both models are not substantially overfitted and support the robustness of our results.

DCA was performed to evaluate the clinical utility of the predictive models across a range of threshold probabilities (**Figure 5**). At low thresholds (< 0.15), the RAR model and the combined model demonstrated nearly identical net benefit. However, across the broader clinically relevant threshold range, the combined model showed superior net benefit compared to RAR alone.

**Figure 5:**
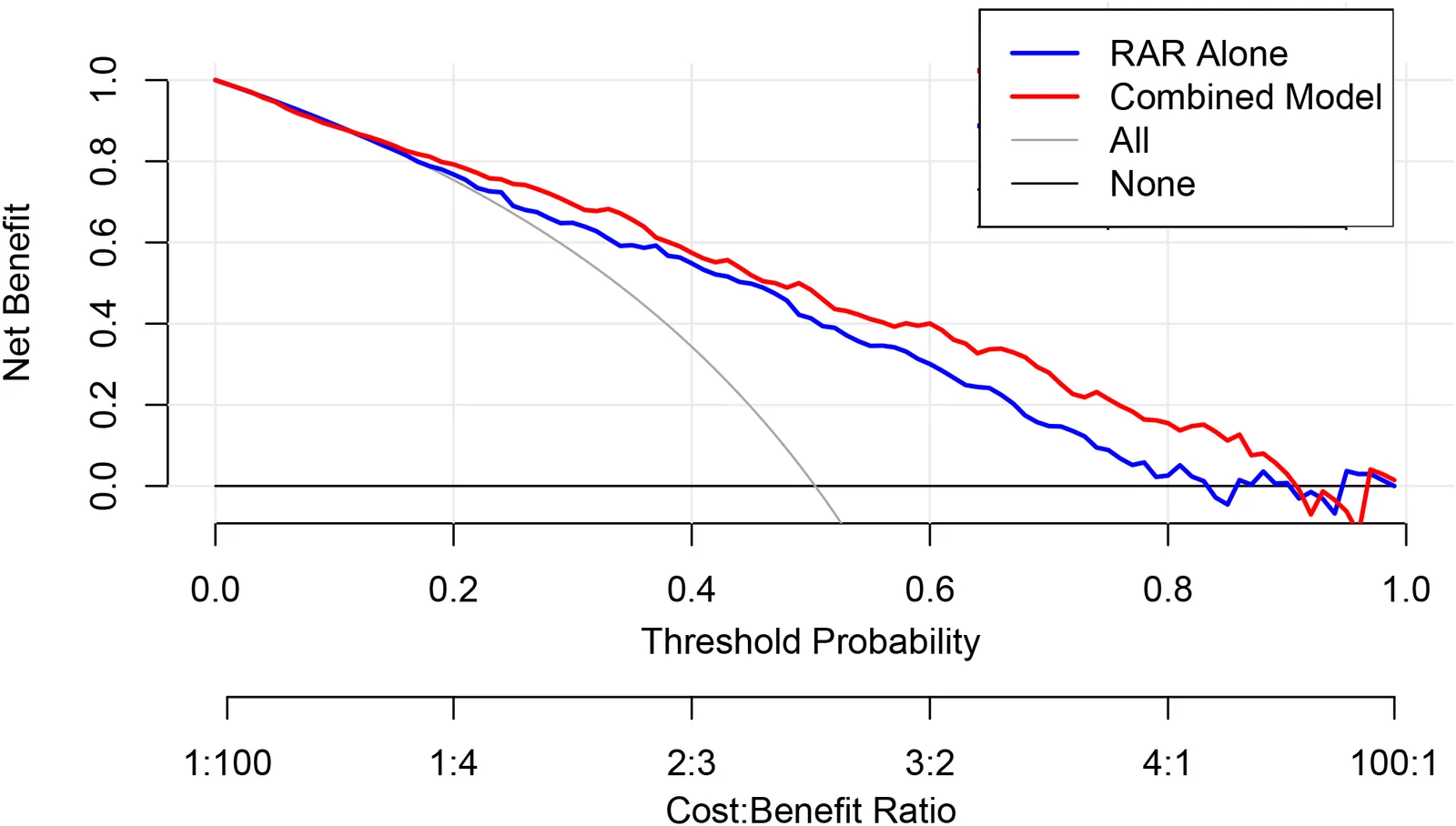
DCA evaluates the clinical net benefit of the models across a range of threshold probabilities.

### 3.4 Exploring the exposure-risk relationship between RAR and the risk of major amputation

We further investigated whether the relationship between RAR and amputation level was linear or nonlinear. The RCS analysis showed that the relationship between RAR and major versus minor amputation was nonlinear (p for nonlinearity =0.014) (**Supplementary Figure S2**). The threshold effects analyses indicated that the inflection point of RAR was 4.84 (**Supplementary Table S1**). When RAR was less than 4.84, the odds of major amputation increased with RAR. Conversely, when RAR exceeded 4.84, the association between RAR and amputation level was not statistically significant.

### 3.5 Exploratory subgroup analysis: above-knee vs. below-knee amputation

Among the 271 patients who underwent major amputation, 55 (20.3%) underwent AKA and 216 (79.7%) underwent BKA (**Supplementary Table S2**). As shown in **Supplementary Table S2**, there was no significant difference in age, sex, BMI, diabetes duration, WBC, neutrophils, CRP, or FBG between the two groups. However, Hb, lymphocytes, monocytes, platelets, albumin, and HbA1c were significantly lower in the AKA group than in the BKA group (all p < 0.05). Among these twelve inflammatory biomarkers, RAR was significantly higher in AKA than in BKA (median [IQR]: 5.63 vs. 4.78, p<0.001). HALP, PNI, ALI and CALLY were significantly lower in AKA than in BKA (median [IQR]: 8.15 vs. 10.28, p=0.046; median [IQR]: 34.50 vs. 37.00, p=0.002; median [IQR]: 6.00 vs. 9.24, p=0.008; median [IQR]: 0.40 vs. 0.57, p=0.030; respectively). Based on these univariate findings and to avoid overfitting due to the limited sample size, RAR, PNI, and ALI were selected for inclusion in the multivariable model, as they represent distinct biological dimensions (inflammation, nutrition, and combined inflammation-nutrition status).

In the multivariable logistic regression model adjusted for age and sex, RAR remained independently associated with AKA (OR = 1.835, 95% CI: 1.224-2.752, p = 0.003). In contrast, PNI (OR = 1.108, 95% CI: 0.642-1.913, p = 0.712) and ALI (OR = 0.646, 95% CI: 0.323-1.291, p = 0.216) were not independently associated with AKA after multivariable adjustment. Age and sex were also not significant predictors in the model (p = 0.467 and p = 0.838, respectively) (**Supplementary Table S3**).

ROC analysis showed that RAR had moderate discriminative ability for distinguishing AKA from BKA (AUC = 0.662, 95% CI: 0.579-0.744, p <0.001). PNI (AUC = 0.632, p = 0.002) and ALI (AUC = 0.616, p = 0.008) showed moderate univariate discriminative ability but were not independently associated with AKA after multivariable adjustment.

## 4 Discussion

This study aimed to comprehensively investigate the associations between a range of inflammation-related biomarkers and amputation level (major versus minor) in patients with DFU who underwent amputation, using data from a single hospital. The results indicated that higher levels of NAR, MAR, RAR, SIRI, SII, AISI, IBI, and CLR were linked to major amputation, whereas lower levels of HALP, PNI, ALI, and CALLY were associated with major amputation. Among these, RAR showed the strongest association with amputation level, and a combined model incorporating RAR, HALP, PNI, and ALI demonstrated superior predictive performance compared to RAR alone (AUC: 0.816 vs. 0.773, p = 0.001). In the exploratory subgroup analysis, RAR, PNI, and ALI differed significantly between the AKA and BKA groups, but only RAR remained independently associated with AKA after multivariable adjustment.

Major lower extremity amputations lead to reduced quality of life and increased healthcare costs. Therefore, identifying factors associated with amputation level in patients with DFUs is a critical issue that guide clinical management. Chronic inflammation and malnutrition are hallmarks of DFU pathogenesis. Specifically, chronic inflammation, characterized by pro-inflammatory cytokines such as TNF-α, IL-1β, and IL-6, impairs angiogenesis and delays wound repair (13). Malnutrition prolongs the inflammatory phase, limits collagen synthesis, and increases the risk of new wound formation (14). Various inflammatory and nutritional indices derived from routine blood tests, which including NAR, MAR, RAR, HALP, PNI, ALI, SIRI, SII, AISI, CALLY, IBI, and CLR, have been established as readily obtainable prognostic markers across diverse diseases (15–25). However, the comparative utility of these indices in differentiating amputation level in DFU patients has not been systematically investigated. Therefore, this study aimed to comprehensively evaluate the associations between these biomarkers and amputation level (major versus minor) in patients with DFU who underwent amputation.

To the best of our knowledge, this is the first study to systematically investigate the association between twelve inflammation related biomarkers and amputation level in patients with DFU undergoing amputation. RAR demonstrated the strongest association with major amputation (OR = 3.25), which remained significant after adjusting for key confounders. RAR integrates RDW, which reflects chronic inflammation, oxidative stress, and poor nutritional status (26), and albumin, a key marker of protein energy malnutrition (27). Indices focusing solely on inflammatory cells or solely on nutritional status may capture only part of the pathological process, whereas RAR encompasses both dimensions. In patients with DFUs, elevated RDW and decreased albumin frequently co-occur due to chronic inflammation and catabolic stress. Consequently, RAR serves as a composite indicator of systemic dysregulation that impairs wound healing and is associated with major amputation level. This finding is consistent with a recent NHANES based study by Huang et al., which demonstrated that RAR was independently associated with DFU in a nationally representative outpatient cohort (28). HALP, which integrates hemoglobin, albumin, lymphocyte, and platelet, was independently associated with minor amputation (OR = 0.34). Patients with low HALP scores are characterized by concurrent malnutrition, anemia, and lymphopenia, all of which impair tissue repair and angiogenesis (29). This finding aligns with the concept that preserving nutritional immune status is critical for limb salvage, and is supported by Li et al., who found that HALP scores are significantly associated with DFU risk (30), and by Yağız et al., who reported that a HALP score below 19.96 was associated with major amputation in DFU patients (31). Of note, PNI showed direction reversal from univariate (OR = 0.47) to multivariable analysis (OR = 3.718), likely reflecting collinearity with other albumin/lymphocyte-based indices. Nevertheless, its inclusion in the combined model significantly improved predictive performance, supporting its value as a contributing factor when interpreted within a multivariable framework.

LASSO regression reduced the twelve indices to eight candidates; subsequent multivariate analysis confirmed that NAR, HALP, ALI, and PNI were independently associated with amputation level. This two-step approach enhances robustness and reduces the risk of false-positive associations. Importantly, the combined model incorporating RAR, HALP, PNI, and ALI demonstrated superior predictive performance compared to RAR alone, with an AUC of 0.816 vs. 0.773 (p = 0.001). This finding suggests that while RAR is a strong individual marker, the addition of HALP, PNI, and ALI provides incremental discriminative value, as each index captures complementary biological dimensions including nutritional-immune status, nutritional reserve, and metabolic-inflammatory balance. Bootstrap internal validation confirmed the stability of the combined model (optimism-corrected AUC: 0.811; optimism: 0.005), ruling out overfitting as an explanation for its superior performance. DCA was performed to evaluate the clinical net benefit of the two models. At low thresholds, the RAR model and the combined model demonstrated nearly identical net benefit. At higher thresholds, the combined model showed a slightly higher net benefit, but the magnitude of this difference was modest. Both models consistently outperformed the ‘treat all’ and ‘treat none’ strategies across the clinically reasonable threshold range, supporting the clinical utility of risk stratification using either model. Therefore, RAR alone may suffice as the preferred tool for risk stratification in resource-limited settings, while the combined model may offer incremental information in selected cases where comprehensive assessment is feasible.

In the exploratory subgroup analysis comparing AKA and BKA among patients undergoing major amputation, RAR was significantly higher in the AKA group, while PNI and ALI were significantly lower. After adjusting for age and sex, only RAR remained independently associated with AKA (OR = 1.835, 95% CI: 1.224-2.752, p = 0.003), suggesting that RAR may help differentiate amputation level among patients already committed to major amputation. The observation that PNI and ALI lost significance in the multivariable model indicates that their univariate associations with AKA were largely captured by RAR, likely reflecting their shared biological pathways involving systemic inflammation and nutritional status. These findings are consistent with the primary analysis, where RAR emerged as the most robust inflammatory marker associated with amputation outcomes.

Previous studies have investigated individual inflammatory biomarkers in DFU. However, most existing research has focused on single indices without comparing a broad panel or exploring subtypes based on amputation level. The present study extends the literature by: (1) systematically comparing twelve indices head-to-head; (2) identifying the RAR as the strongest individual predictor; (3) demonstrating that a combined model incorporating RAR, HALP, PNI, and ALI provides superior predictive performance compared to RAR alone; and (4) revealing distinct inflammatory profiles for AKA versus BKA. Our findings have several practical implications. For risk assessment in patients undergoing amputation: RAR can serve as a rapid screening tool. A practical cut-off value (e.g., RAR > 4.312) may alert clinicians to higher likelihood of major amputation, prompting early vascular referral or intensified infection control. For improved risk stratification: the combined model offers incremental discriminative value, supporting its use in situations where more comprehensive risk assessment is needed. For nutritional interventions: The identification of HALP as a protective factor associated with minor amputation underscores the importance of nutritional assessment. Patients with low HALP may benefit from aggressive nutritional support, although whether this can improve outcomes requires prospective investigation. For amputation level planning: Among patients requiring major amputation, elevated RAR may help identify those at risk for failed BKA, supporting consideration of primary AKA.

Several limitations should be acknowledged. First, due to the retrospective design, we were unable to obtain detailed clinical data on several major determinants of amputation level, including Wagner grade, comprehensive peripheral arterial disease assessment, osteomyelitis, gangrene extent, and revascularization status. While we adjusted for available demographic and metabolic factors (age, sex, FBG, BMI, hypertension, and diabetes duration), we cannot exclude. The absence of these variables may lead to overestimation of the observed associations. The associations of pro-inflammatory markers may be overestimated, while the protective effect of HALP may be underestimated. The absence of revascularization history is a particularly important limitation, as prior revascularization procedures directly influence both the decision and the level of amputation. Similarly, infection severity and osteomyelitis extent are critical determinants of amputation outcomes. These findings should be interpreted as hypothesis-generating, and causality cannot be inferred. Second, the single-center design limits the generalizability of our findings. Although amputation decisions at our institution were made by a multidisciplinary team following standardized WIfI-based criteria, surgeon preferences, institutional practices, and local resource availability vary considerably across centers. Such variation in clinical decision-making could affect the composition of the amputation groups and, consequently, the observed associations between biomarkers and amputation level. Therefore, our findings should be extrapolated to other populations with caution, and external validation in multicenter cohorts with different decision-making practices is needed. Third, our study included only patients who had already undergone amputation; therefore, our findings apply to the comparison between major and minor amputation among patients with established surgical indication, rather than to the prediction of future amputation risk in the general DFU population. Fourth, all indices were measured at a single time point (admission); dynamic changes during treatment were not captured. Future studies should investigate whether serial measurements improve discriminative ability. Fifth, the AKA subgroup analysis was limited by the small sample size (n = 55). To minimize overfitting, we included only five covariates (age, sex, RAR, PNI, and ALI), yielding approximately 11 events per variable. Nevertheless, these results should be considered hypothesis-generating rather than confirmatory, and validation in larger cohorts is needed. Sixth, minor amputation encompasses a heterogeneous group of procedures (toe to midfoot) with varying functional outcomes. Detailed subclassification of these types was not available in our retrospective dataset. We acknowledge that grouping them together may introduce heterogeneity and dilute the true associations. Future studies should collect granular data on amputation subtypes to enable separate analyses. Finally, the optimal cut-off values for RAR and other indices are unlikely to be stable or generalizable and serve here primarily as an initial clinically intuitive reference, our findings regarding specific cutoffs are strictly preliminary and must be validated in larger, prospective studies before any clinical application can be considered.

## 5 Conclusion

In conclusion, this study systematically evaluated twelve inflammation-related biomarkers in diabetic foot ulcer patients undergoing amputation. Among those, RAR showed the strongest association with major versus minor amputation. A combined model incorporating RAR, HALP, PNI, and ALI demonstrated superior predictive performance compared to RAR alone, but the incremental clinical gain was modest. In the exploratory subgroup analysis of patients requiring major amputation, RAR was associated with AKA versus BKA after multivariable adjustment, whereas PNI and ALI were not significant. These findings suggest that RAR alone may serve as a simple and cost-effective tool for risk stratification in routine practice, particularly in resource-limited settings, while the combined model may offer marginal additional information when comprehensive assessment is feasible. However, clinical translation requires prospective validation.

## Data Availability

The original contributions presented in the study are included in the article/**Supplementary Material**. Further inquiries can be directed to the corresponding author.

## Supplementary material

Supplementary Figure 1 Calibration curve. **(A)** Combined model; **(B)** RAR alone model.

Supplementary Figure 2 RCS analysis of the association between RAR and major versus minor amputation.
