# Supplementary material for: Association of novel inflammatory biomarkers with major amputation level in diabetic foot ulcer patients undergoing amputation: a retrospective comparative study
Source: Front Endocrinol (Lausanne). 2026 Sep 21;17:1925839. doi: 10.3389/fendo.2026.1925839 (PMC13635333; doi:10.3389/fendo.2026.1925839)
Supplement: Supplementary file 1 [file Table1.doc]

Table S1 threshold effect analysis RAR on the risk of major amputation

| Outcome | effect | *P* |
| --- | --- | --- |
| Model 1 Fitting model by standard linear regression | 2.76 (2.23 - 3.41) | <0.001 |
| Model 2 Fitting model by two-piecewise linear regression |  |  |
| Inflection point | 4.84 |  |
| <5.58 | 4.62 (2.78 - 7.65) | <0.001 |
| ≥5.58 | 1.53 (0.94 - 2.50) | 0.085 |
| P for likelihood test |  | 0.011 |

Table S2 Clinical characteristics of AKA vs. BKA

| Characteristics | AKA (n=55) | BKA (n=216) | p |
| --- | --- | --- | --- |
| Age (years) | 65.75 ± 10.65 | 65.55 ± 10.84 | 0.903 |
| Gender |  |  |  |
| Male | 40 | 162 | 0.730 |
| Female | 15 | 54 |  |
| Hypertension |  |  |  |
| Yes | 26 | 120 | 0.707 |
| No | 29 | 96 |  |
| BMI (kg/m2) | 21.90 ± 3.06 | 22.50 ± 3.48 | 0.248 |
| Duration of diabetes (years) | 18.96 ± 4.69 | 18.92 ± 4.40 | 0.950 |
| CRP (mg/L) | 91.08 ± 69.63 | 79.09 ± 57.11 | 0.186 |
| Hb (g/L) | 84.44 ± 21.82 | 92.81 ± 19.47 | 0.006 |
| WBC (10^9^) | 14.11 ± 8.93 | 13.41 ± 6.53 | 0.518 |
| Lymphocyte (10^9^) | 1.17 ± 0.59 | 1.39 ± 0.63 | 0.020 |
| Monocyte (10^9^) | 0.69 ± 0.38 | 0.85 ± 0.42 | 0.012 |
| Neutrophil (10^9^) | 11.87 ± 8.11 | 10.85 ± 6.16 | 0.306 |
| Platelet (10^9^) | 302.31 ± 125.48 | 353.37 ± 131.16 | 0.010 |
| RDW (%) | 15.43 ± 2.51 | 14.37 ± 2.08 | 0.001 |
| Albumin (g/L) | 28.11 ± 5.18 | 30.08 ± 5.04 | 0.011 |
| FBG (mmol/L) | 9.82 ± 4.31 | 9.80 ± 4.21 | 0.975 |
| HbAlc (%) | 7.86 ± 1.98 | 8.63 ± 2.03 | 0.012 |
| NAR | 3.55 (2.21, 5.78) | 3.02 (1.96, 4.90) | 0.220 |
| MAR | 0.24 (0.16, 0.30) | 0.25 (0.19, 0.35) | 0.108 |
| RAR | 5.63 (4.43, 6.55) | 4.78 (4.08, 5.66) | <0.001 |
| HALP | 8.15 (5.07, 13.53) | 10.28 (6.51, 16.38) | 0.046 |
| PNI | 34.50 (29.05, 39.00) | 37.00 (33.30, 41.42) | 0.002 |
| ALI | 6.00 (3.71, 11.35) | 9.24 (4.94, 15.55) | 0.008 |
| SIRI | 5.25 (3.01, 11.32) | 5.35 (2.92, 11.46) | 0.929 |
| SII | 3346.40 (1803.93, 4886.44) | 2498.94 (1313.51, 4215.46) | 0.239 |
| AISI | 1614.47 (923.89, 3048.04) | 1914.64 (858.30, 3803.34) | 0.443 |
| CALLY | 0.40 (0.20, 0.85) | 0.57 (0.28, 1.29) | 0.030 |
| IBI | 609.62 (210.40, 1782.04) | 448.09 (173.98, 1237.61) | 0.086 |
| CLR | 63.13 (35.86, 121.73) | 53.55 (23.18, 91.21) | 0.057 |

AKA: above-knee amputation; BKA: below-knee amputation; RDW, red cell distribution width; FBG: Fasting Blood Glucose; NAR, neutrophil-albumin ratio; MAR, monocyte-albumin ratio; RAR, red cell distribution width-albumin ratio; HALP, hemoglobin, albumin, lymphocyte, and platelet; PNI, prognostic nutritional index; ALI: advanced lung cancer inflammation index; SIRI: systemic inflammatory response index; SII: systemic immune-inflammatory index; AISI: aggregate index of systemic inflammation; CALLY: C-reactive protein-albumin-lymphocyte index; IBI: :inflammatory burden index; CLR: C-reactive protein to lymphocyte ratio

Table S3 Multivariable regression for AKA vs BKA

| Variable | β coefficient | Standard Error | P value | OR | 95% CI |
| --- | --- | --- | --- | --- | --- |
| Age | 0.011 | 0.015 | 0.467 | 1.011 | 0.981-1.042 |
| Sex | -0.074 | 0.362 | 0.838 | 0.929 | 0.456-1.889 |
| **ZRAR** | **0.607** | **0.207** | **0.003** | **1.835** | **1.224-2.752** |
| ZPNI | 0.103 | 0.278 | 0.712 | 1.108 | 0.642-1.913 |
| ZALI | -0.437 | 0.353 | 0.216 | 0.646 | 0.323-1.291 |
| Constant | -2.562 | 1.113 | 0.021 | 0.077 | — |

RAR, red cell distribution width-albumin ratio; PNI, prognostic nutritional index; ALI: advanced lung cancer inflammation index;
